# Transient Focal Cerebral Ischemia/Reperfusion Induces Early and Chronic Axonal Changes in Rats: Its Importance for the Risk of Alzheimer's Disease

**DOI:** 10.1371/journal.pone.0033722

**Published:** 2012-03-23

**Authors:** Qinan Zhang, Teng Gao, Yi Luo, Xijuan Chen, Ge Gao, Xiaoqun Gao, Yiwu Zhou, Jiapei Dai

**Affiliations:** 1 Wuhan Institute for Neuroscience and Neuroengineering, South-Central University for Nationalities, Wuhan, China; 2 Department of Anatomy, Medical College of Zhengzhou University, Zhengzhou, China; 3 Brain Paralysis Research Center, Medical College of Zhengzhou University, Zhengzhou, China; 4 Department of Forensic Medicine, Tongji Medical College of Huazhong University of Science & Technology, Wuhan, China; Nathan Kline Institute and New York University School of Medicine, United States of America

## Abstract

The dementia of Alzheimer's type and brain ischemia are known to increase at comparable rates with age. Recent advances suggest that cerebral ischemia may contribute to the pathogenesis of Alzheimer's disease (AD), however, the neuropathological relationship between these two disorders is largely unclear. It has been demonstrated that axonopathy, mainly manifesting as impairment of axonal transport and swelling of the axon and varicosity, is a prominent feature in AD and may play an important role in the neuropathological mechanisms in AD. In this study, we investigated the early and chronic changes of the axons of neurons in the different brain areas (cortex, hippocampus and striatum) using in vivo tracing technique and grading analysis method in a rat model of transient focal cerebral ischemia/reperfusion (middle cerebral artery occlusion, MCAO). In addition, the relationship between the changes of axons and the expression of β-amyloid 42 (Aβ42) and hyperphosphorylated Tau, which have been considered as the key neuropathological processes of AD, was analyzed by combining tracing technique with immunohistochemistry or western blotting. Subsequently, we found that transient cerebral ischemia/reperfusion produced obvious swelling of the axons and varicosities, from 6 hours after transient cerebral ischemia/reperfusion even up to 4 weeks. We could not observe Aβ plaques or overexpression of Aβ42 in the ischemic brain areas, however, the site-specific hyperphosphorylated Tau could be detected in the ischemic cortex. These results suggest that transient cerebral ischemia/reperfusion induce early and chronic axonal changes, which may be an important mechanism affecting the clinical outcome and possibly contributing to the development of AD after stroke.

## Introduction

Alzheimer's disease (AD) and vascular dementia (VaD) are widely accepted as the most common forms of dementia and demonstrate similar increases with age [1], [2]. AD is a severe neurodegenerative disorder defined histologically by the presence of senile plaques (SPs) containing β-amyloid (Aβ), and neurofibrillary tangles (NFTs). In previous studies, cerebral ischemia has been generally considered an exclusion criterion for clinical diagnosis of AD [3], however, recent advances in the epidemiology of AD reveal that ischemic disease significantly affects 60% to 90% of patients with AD and increases the risk of developing AD [4]–[6]. It is estimated that AD is three times more likely to precipitate in old age after a transient ischemia attack (TIA) [2], and it is clear that cerebral ischemia may exacerbate dementia and worsen outcomes in AD patients [4], [6], [7]. There are several isolated reports suggesting an interaction between AD and cerebral ischemia [2], [4], [8], [9], nevertheless, we have not yet understood the processes and nature of the acute ischemic events triggering the onset and progression of AD.

Various hypotheses regarding the causes of AD have been debated, and there is currently no consensus on this issue [10]–[13]. An increasing body of evidence has implicated that axonopathy, mainly manifesting as impairment of axonal transport and swelling of the axon and varicosity, may play an important role in the etiology of AD. Such changes have been identified in living AD patients, in postmortem AD brains and in different animal models [14]–[20], however, so far no study has investigated long-term axonal changes after transient focal cerebral ischemia.

To explore the importance of cerebral ischemia for the risk of AD, we observed the morphological changes of axons of neurons in the different brain areas (cortex, hippocampus and striatum) following transient focal cerebral ischemia/reperfusion in rats. In addition, we determined whether axonal changes after transient focal cerebral ischemia/reperfusion were related to AD-associated neuropathological changes such as SPs and NFTs.

## Results

### Transient focal cerebral ischemia/reperfusion induced early and chronic axonal changes

**Figure 1:**
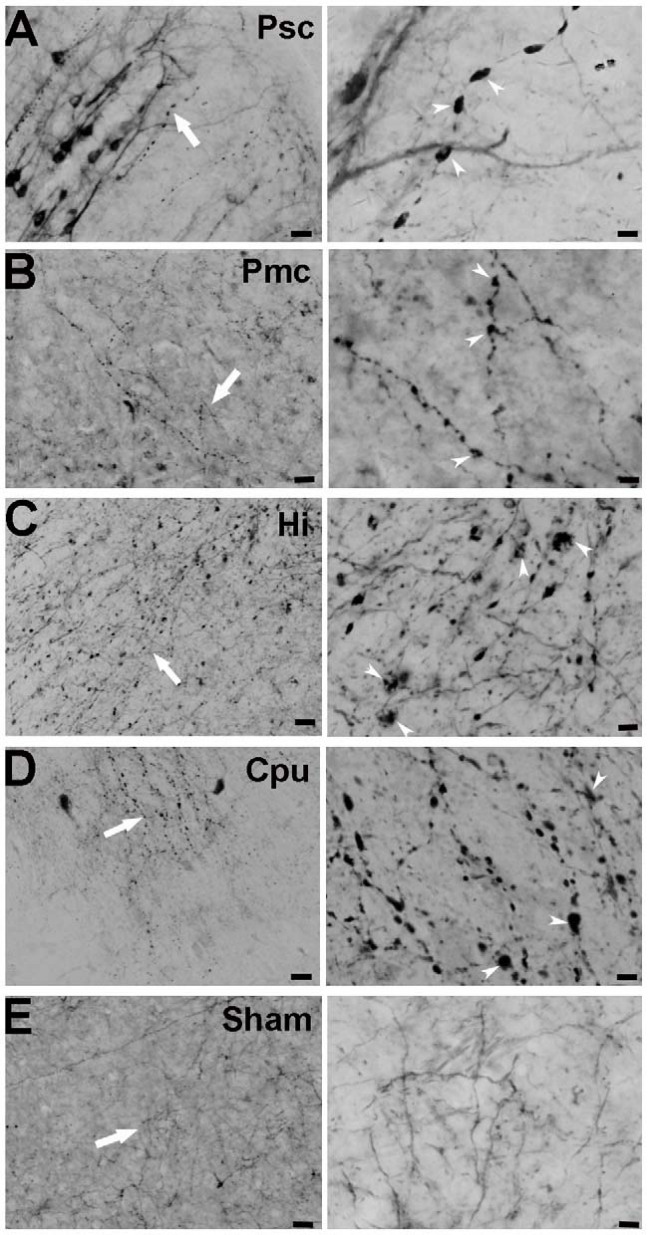
Axonal morphological changes demonstrated by in vivo tracing in the different brain areas after transient cerebral ischemia/reperfusion. Right panels were high magnification views of the areas in left panels in A–E (single arrow), respectively. Typical swollen axons or varicosities (arrowheads) could be seen in the primary sensory cortex (Psc) (A), primary motor cortex (Pmc) (B), hippocampus (Hi) (C) and caudoputamen (Cpu) (D) of the ischemic hemisphere after transient cerebral ischemia/reperfusion. No obvious axonal changes were noticed in the primary sensory cortex from the sham group (E). Scale bars: 20 µm for left panels and 5 µm for right panels in A–E.

**Figure 2:**
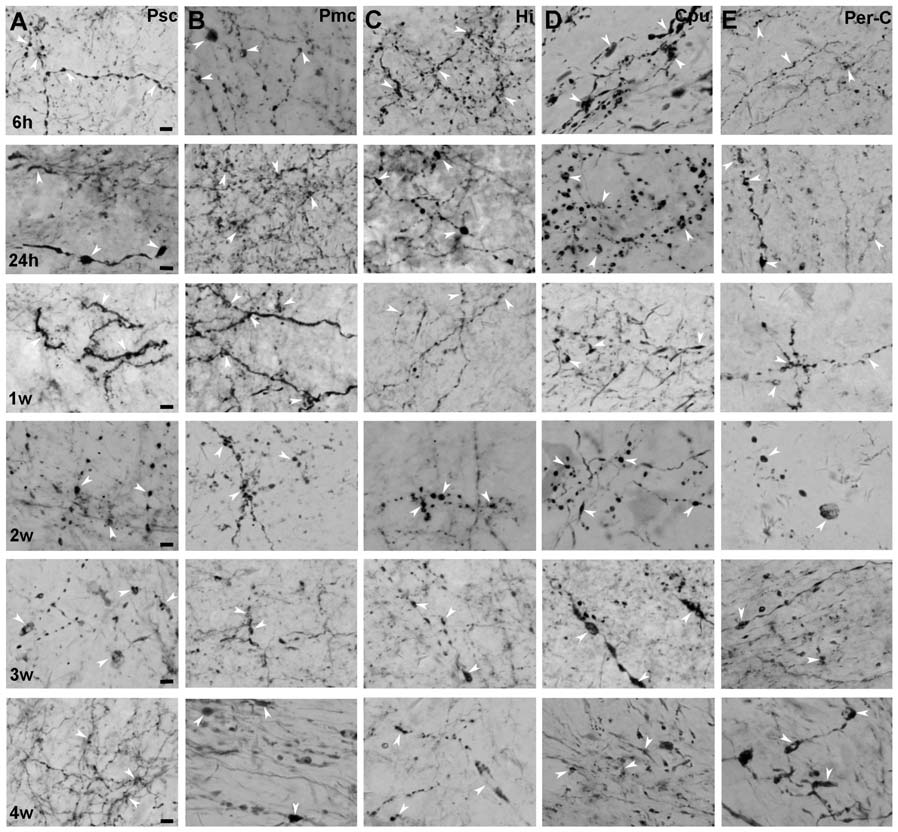
Transient cerebral ischemia/reperfusion induces early and chronic axonal changes. Representative axonal changes (swellings of axons and varicosities) can be seen at 6 h, 24 h, 1 w, 2 w, 3 w and 4 w after transient cerebral ischemia/reperfusion in different brain regions, including the ischemic primary sensory cortex (Psc) (A), primary motor cortex (Pmc) (B), hippocampus (Hi) (C), caudoputamen (Cpu) (D) and peri-lesional cortex (E). Arrowheads in each figure mark the swollen axons or varicosities. Scale bars: 5 µm for A–E.

Using in vivo tracing, we examined the effects of transient focal cerebral ischemia/reperfusion on axonal changes in the ischemic or perilesional cortex, caudoputamen (striatum) and hippocampus of the MCAO hemisphere. The typical axonal changes presenting swollen axons and varicosities were detected in the different brain areas (Fig. 1A–D). Swollen axons and varicosities appeared in the ischemic sensory and motor cortex, perilesional cortex, hippocampus and caudoputamen as early as 6 h (Fig. 2A–E) after transient cerebral focal ischemia/reperfusion as compared to the corresponding contralateral regions and the sham-operated group (Fig. 1E). Such axonal changes even extended to 4 w after transient cerebral focal ischemia/reperfusion (Fig. 2A–E).

**Figure 3:**
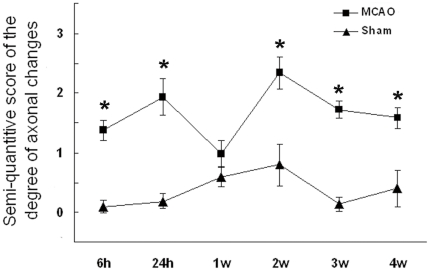
Semi-quantitative score of the degree of axonal changes in ischemic brain regions of transient cerebral ischemia/reperfusion and control rats from axonal tracing evaluation. The degree of swollen axons and varicosities was particularly obvious at 2 w after transient cerebral ischemia/reperfusion. Such axonal damages could extend to 4 w as compared to the corresponding contra-lateral brain regions in sham groups, although appeared to self-recover temporarily at 1 w. The score at each time point represents 13–19 values (Mean±SD) obtained from 3–4 rats in each subgroup. The asterisk indicates a highly significant difference (P<0.01) between the transient cerebral ischemia/reperfusion subgroup and the corresponding sham subgroup.

We determined the degree of axonal changes from axonal tracing evaluation using a grade system and found that the degree of swollen axons and varicosities was particularly obvious (similar to grade 2) at 2 w after transient cerebral ischemia/reperfusion (Table 1, Fig. 3) and extended to 4 w, although such axonal damages appeared to recover gradually after 2 w. Unexpectedly, obvious changes found at 24 h seemed to self-recover temporarily at 1 w after transient cerebral ischemia/reperfusion compared to the corresponding sham-operated group (Table 1, Fig. 3).

**Table 1:** Semi-quantitative analysis of the degree of axonal changes in different brain regions in transient cerebral ischemia/reperfusion and control rats from axonal tracing evaluation.

MCAO	Case No	Psc	Pmc	Per- C	Hi	Cpu	Sham	Case No	Psc	Pmc	Per- C	Hi	Cpu
6 h	N1	2	3	1	1	1	6 h	N1	1	0	0	0	0
	N2	2	1	2	0	2		N2	0	0	0	0	0
	N3	1	1	1	2	ND		N3	0	0	0	0	ND
	N4	1	2	0	2	ND		N4	0	0	0	1	0
24 h	N1	2	ND	3	2	1	24 h	N1	0	0	0	1	ND
	N2	3	2	1	2	3		N2	0	0	0	ND	1
	N3	1	2	1	2	2		N3	1	0	0	0	ND
								N4	0	0	0	0	0
1 w	N1	1	2	1	1	1	1 w	N1	0	1	0	ND	1
	N2	1	1	0	2	ND		N2	1	1	0	1	1
	N3	1	1	0	ND	1		N3	1	ND	0	0	1
2 w	N1	2	2	2	3	ND	2 w	N1	1	1	1	1	1
	N2	2	3	2	ND	3		N2	1	1	1	1	1
	N3	2	2	2	2	2		N3	1	0	0	0	1
	N4	2	3	2	3	3							
3 w	N1	2	2	0	2	ND	3 w	N1	0	0	0	1	0
	N2	2	1	2	3	1		N2	0	0	0	1	0
	N3	1	2	1	2	3		N3	0	0	0	0	0
	N4	2	2	1	1	3							
4 w	N1	1	2	2	1	1	4 w	N1	0	ND	0	1	0
	N2	ND	2	1	2	2		N2	0	0	0	1	0
	N3	2	2	1	2	1		N3	1	1	0	ND	1

The numbers (1–3) represent the degree of axonal changes (see also in method). MCAO: middle cerebral artery occlusion and reperfusion; Psc: primary sensory cortex; Pmc: primary motor cortex; Hi: hippocampus; Cpu: caudoputamen; Per-C: perilesional cortex; ND: not detected.

### Lack of Aβ plaques or overexpression of Aβ42 after transient focal cerebral ischemia/reperfusion

Assemblies of Aβ42 are believed to contribute to the formation of senile plaques, one of the classic neurodegenerative changes in AD. To investigate the relationship between the axonal changes induced by cerebral ischemia and the formation and development of Aβ plagues, we used double immunohistochemical staining for tracer and Aβ42. We did not observe the classic Aβ plaques and overexpression of Aβ42 within and/or close to the swollen axons and varicosities in the ischemic brain regions at each time point after reperfusion (Figure not shown).

### Aberrant Tau hyperphosphorylation occurs in the ischemic cortex

**Figure 4:**
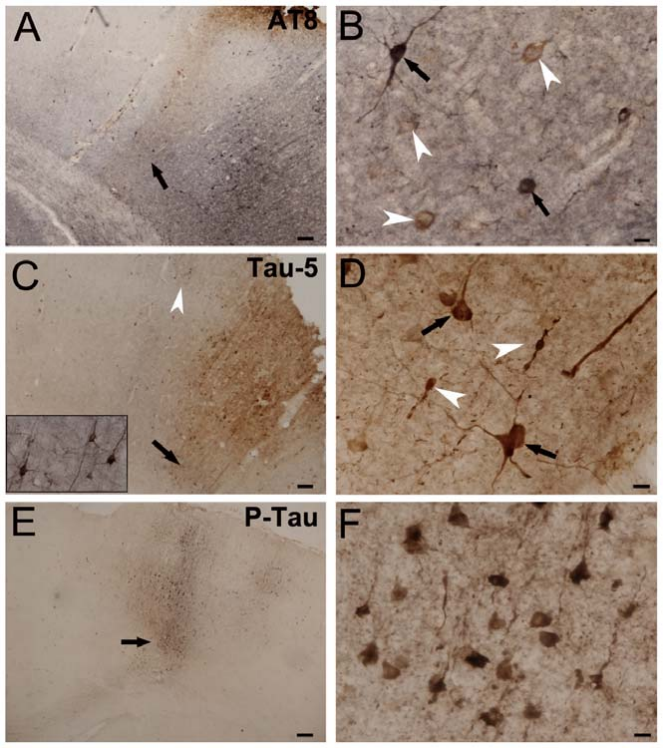
Tracing and immunohistochemical double staining show the tracer-labeled neurons and the expression of hyperphosphorylated Tau induced by transient cerebral ischemia/reperfusion. B, D and F are the magnification of an area in A, C and E, respectively. Some staining for AT8 (brown color) was found in the ischemic cortex (A) and AT8 labeled neuronal bodies could be noticed (B, arrowheads). The arrows in B indicate the tracer-labeled neurons (dark blue color). Strong staining for Tau-5 (brown color) was found in the ischemic cortex (C) and the extensive Tau-5 labeled neurons (D, arrows) and swollen axons (D, arrowheads) were noticed. Inserted figure in C showed the magnification of an area (arrowhead), demonstrating the tracer-labeled neurons (dark blue color). Only very weak staining could be observed for P-tau (E) and tracer-labeled neurons presented the morphological changes as cell shrinkage occurred. Scale bars: 10 µm for B, D, and F, 100 µm for A and C, 200 µm for E.

To characterize the phosphorylation status of Tau in the ischemic regions, double immunohistochemical staining for tracer and phosphorylated Tau protein (P-tau and AT8) or the total Tau (Tau-5) was performed. It was found that only some Tau protein staining for AT8 and Tau-5 could be detected within the ischemic and perilesional cortex (Fig. 4A and B), whereas the staining for P-tau was very weak (Fig. 4C).

**Figure 5:**
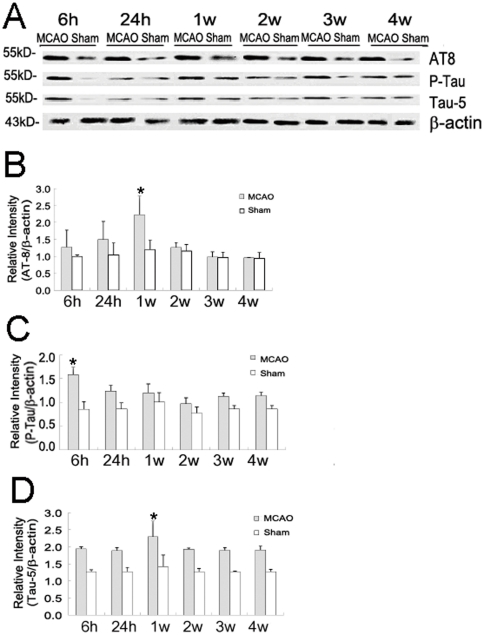
Western blot testing shows the expression of hyperphosphorylated Tau induced by transient cerebral ischemia/reperfusion. The level of Tau protein increased in the ischemic cortex of the transient cerebral ischemia/reperfusion subgroup compared to the corresponding region in the sham group (A through D). The levels of AT8 and Tau-5 increased obviously at 1 w after transient cerebral ischemia/reperfusion (B and D), whereas a similar increase was detected in the level of P-tau only at 6 h after transient cerebral ischemia/reperfusion (C). The asterisk indicates a significant difference (P<0.05) between this subgroup and the other five subgroup.

To further explore the detailed effect in the cortex of cerebral ischemia on Tau phosphorylation over time, we used protein immunoblotting for quantitative analysis and found that there was an enhancement of Tau phosphorylation at 6 h after reperfusion (Fig. 5A). The level of AT8 was increased significantly in the ischemic cortex at 1 w as compared to the ipsilateral side of ischemic cortex at 6 h, 24 h, 2 w, 3 w or 4 w, respectively (Fig. 5A and B). Whereas the level of P-tau was significantly increased only 6 h after transient cerebral ischemia/reperfusion (Fig. 5A and C). Similar to AT8, the total amount of Tau detected with Tau-5 reached the peak at 1 w after transient cerebral ischemia/reperfusion (Fig. 5A and D).

## Discussion

In the present study, using an in vivo tracing technique, we could observe the development process of the morphological changes of axons after transient cerebral ischemia/reperfusion, mainly manifesting as axonal swellings or spheroids, which were similar to the axonopathy in AD brains and in animal models of AD [14]–[20]. Even 6 h after the reperfusion, the swellings of axons and varicosities had obviously appeared in the ischemic and perilesional regions, including the sensory and motor cortex, hippocampus and striatum. Axonal damages did not increase gradually after transient cerebral ischemia/reperfusion, however, such axonal changes could exist for up to 4 w after reperfusion. To our surprise, no obvious changes were found at 1 w after transient cerebral ischemia/reperfusion compared to the corresponding sham-operated group, the underlying mechanisms are not clear and need to be further elucidated.

As an acute neurodegenerative and dementia-causing disorder in the elderly, ischemic stroke has been recognized as contributing to the pathogenesis of AD [1], [8], [21]. Nevertheless, the pathological mechanism of AD induced by cerebral ischemia seems poorly understood. Recent advances suggest that axonopathy may play a key role in the initiation of the neuropathological processes and cognitive defects in AD. For example, axonopathy has been identified in mouse models of AD preceding known disease-related pathology, and it is considered as an early stage of AD [15]. Axonal transport impairment and the swellings of the axons and varicosities have also been observed both in living AD patients and in other animal models [14]–[20]. In the current study, such axonal changes were detected in several ischemic or perilesional regions, including sensory and motor cortex, hippocampus and striatum, suggesting that axonopathy may be an important neuropathological change linking cerebral ischemia with AD.

The classic pathological hallmarks of AD are SPs and NFTs, which are potentially linked to alterations of the axonal compartment [22]. Although the formation and development of SPs and NFTs are two defining characteristics of neuropathological processes in AD, a debate still persists over whether SPs or NFTs play key and causal roles in the neuropathological mechanisms of AD [10]–[13], and a proposal has been raised that the formation of Aβ plaques and hyperphosphorylated Tau may be the consequences of axonopathy [9], [15], [20]. In the present study, we tested the expression of Aβ42 and Tau hyperphosphorylation by using double immunohistochemical staining for tracer and Aβ42 or Tau protein (P-tau, Tau-5, AT8) in order to study the possible relationship between AD and cerebral ischemia. Consequently, we did not observe Aβ plaques or overexpression of Aβ42 in the ischemic and perilesional brain areas, but found weak staining against hyperphosphorylated Tau (AT8 or P-tau) or the total Tau (Tau-5), accompanied by a few tracer-labeled fibers observed in the ischemic cortex. In addition, we performed a quantitative analysis for Tau protein in ischemic cortex over time after transient cerebral ischemia/reperfusion using western blotting and found significant increases in the total Tau and hyperphosphorylated Tau (pS202 and pS199), suggesting that a specific set of kinases were activated during the short period after reperfusion, which may contribute to the formation of intracellular Tau aggregates. Moreover, our study demonstrated no significant increase in Tau protein expression in the ischemic caudoputamen, suggesting that the lesions restricted to the caudoputamen were different from the lesions in the ischemic cortex.

In conclusion, our data have demonstrated that the swellings of axons and varicosities occurred early and could exist for up to 4 w after transient cerebral ischemia/reperfusion accompanied by weak Tau expression, but without the over-expression of β-amyloid and amyloid deposition, which may be important for understanding the role of ischemic stroke in the pathogenesis of AD. It has been found that patients with cerebrovascular disease often show AD pathology at autopsy, even if there is no hint or clinical expression of pre-existing AD [21]. Thus, we propose that cerebral ischemia that induces axonal changes, including the swellings of axons and varicosities and impaired axonal transport, may be an important mechanism affecting the clinical outcome and possibly contributing to the development of AD after stroke.

## Materials and Methods

### Rat model of transient focal cerebral ischemia/reperfusion

3-month-old male Wistar rats weighing 280–320 g (from the Experimental Animal Center of Hubei Province, Wuhan, China) were housed in a room with a 12-hour light/dark cycle (lights on at 7:00 AM) with access to food and water ad libitum throughout the experimental period. All animal experiments were conducted with the approval of the Animal Care Committee of South-Center University for Nationalities.

The animals were anesthetized with 10% chloral hydrate (0.3 ml/100 g) and subjected to either middle cerebral artery occlusion (MCAO) or sham operation. The right middle cerebral artery (MCA) was occluded using the intraluminal suture technique described previously [23]. Briefly, the right carotid region was exposed through a midline cervical incision, the external carotid was ligated with a 4-0 suture and the common carotid artery was blocked with an artery clip. A 4-0 monofilament nylon suture, whose tip had been rounded by heating, was introduced from the carotid bifurcation into the internal carotid artery until a resistance was encountered (18±0.5 mm), thereby occluding the origin of the MCA. After 30 min, recirculation was established by gentle withdrawal of the suture. For the rats in the sham group, the right carotid region was only exposed but not cut. The body temperature of rats was maintained in the normal range during surgery using a heating lamp. The rats in the MCAO group and the sham-operated group were assigned to six subgroups according to the time of sacrifice (6 h, 24 h, 1 w, 2 w, 3 w and 4 w after surgery).

### Neurological assessment

Neurological deficit was evaluated after surgery by Zea Longa test on a four-point scale (0 = no deficit, 1 = failure to extend right forepaw fully, 2 = circling to the right, 3 = falling to the right, and 4 = no spontaneous walking with a depressed level of consciousness) [23]. Rats scoring 1–3 points indicated successful model establishment.

### In vivo tracing

All rats were subjected to in vivo tracing 3–5 days before sacrifice at the desired time point on 1 w, 2 w, 3 w and 4 w (four subgroups) with exception of other two subgroups, in which the tracing was executed 3 days before MCAO and the animals were killed at 6 h and 24 h after MCAO, respectively. For in vivo tracing, animals were anesthetized with 10% chloral hydrate (0.3 ml/100 g) and mounted with their heads in a standard stereotaxic apparatus (Stoelting, USA) before receiving tracer injection [10% biotinylated dextran amine (BDA) in 0.05 M TBS (0.05 M Tris, 0.9% NaCl, pH 7.6), molecular weight 10,000, Molecular Probes, Invitrogen] under pressure (Microsyringe pump controller, WPI, USA), using a glass pipette with a tip of maximally 40–50 µm in diameter; the glass pipette was filled with an injection volume of 15 nl. All coordinates for the injected regions were adapted from the Rat Brain in Stereotaxic Coordinates (by George Paxinos and Charles Watson). The tracer was injected into the motor cortex (bregma 0 mm, lateral 2 mm, ventral 2 mm), the sensory cortex (bregma −2 mm, lateral 5 mm, ventral 3 mm), the hippocampus (bregma −4 mm, lateral 3 mm, ventral 3 mm) and the striatum (bregma 1 mm, lateral 3.5 mm, ventral 5 mm) of the MCAO hemisphere. The injection sites in the cortex were located at the ischemic area or in part of the perilesional cortex. In addition, the tracer was injected into the contralateral cortex for comparison in one or two animals in each subgroup in order to observe the possible damage effects of glass pipette during the tracer injection.

### Tracer detection and immunohistochemistry

After tracer injection, the animals were allowed to survive for 3–5 days in the same housing conditions. Then animals were deeply anesthetized with 10% chloral hydrate and perfused with saline, followed by a solution of 4% paraformaldehyde in 0.1 M PBS (pH 7.4) at 4°C. The brains were removed and kept in the same fixative at 4°C for overnight postfixation, equilibrated 48 h with 30% sucrose in 0.1 M PBS. Brains were coronally cut in a cryostat into 30 µm sections, and then sections were collected in sequential order in six vials and rinsed in 0.05 M TBS. Sections were treated with 3% H_2_O_2_ for 10 min and rinsed in 0.05 M TBS for 30 min, and then sections were stored for further experiments in 50% glycerol in 0.05 M TBS in refrigeration (4°C). One vial was used to perform tracer staining, four vials for double staining and remaining one vial for reservation.

For tracer detection, the sections from in vivo tracing were incubated with the Avidin-Biotin Complex (Vector Laboratories, Burlingame, CA, USA, 1∶800) in a mixture of 0.05 M Tris, 0.9% NaCl, 0.25% gelatin, and 0.5% Triton-X 100, pH 7.4 for 2 h at room temperature. After several rinses with TBS, the sections were incubated with 0.05% 3,3′-diaminobenzidine tetrahydrochloride (DAB), 0.2% nickel ammoniumsulphate, and 0.003% H_2_O_2_ in 0.05 M TBS (pH 7.4), mounted on gelatin-coated slides, and then dehydrated, cleared, and coverslipped.

The double staining combining tracer detection with immunocytochemistry was carried out following the procedures described in detail in a previous article [20]. After visualization of the tracer with DAB + nickel ammoniumsulphate, some sections were processed for immunocytochemical detection of Aβ42 or hyperphosphorylated Tau (P-tau, Tau-5 and AT8) with the ABC Elite detection method (Vector; Burlingame, CA, USA). Polyclonal antibody against Aβ42 (1∶500) was produced in rabbit by immunization with a synthetic peptide corresponding to residues Aβ42 of human (Bioss, Beijing, China; catalog no: Bs-0107R). This antibody was tested to identify the Aβ deposits and plaques in the brain sections from AD brains [20], rat and mouse. Rabbit polyclonal antibody P-tau (1∶5000) recognizing Tau when serines 199 and 202 are phosphorylated (Invitrogen, USA; catalog no: 44–768G). Tau-5 (1∶5000) monoclonal antibody reacting with non-phosphorylated Tau and the phosphorylated form of Tau (Invitrogen, USA; catalog no: AHB0042). Antihuman PHF-tau monoclonal antibody (AT8) reacting with an epitope including phosphorylated Ser202 and Thr205 residues (1∶10, 000; Pierce; Appleton, WI, USA; catalog no: MN1020). AT8 can stain pre-tangles, NFTs and extracellular tangles due to the recognition of hyperphosphorylated tau according to the previous report [24].

### Semi-quantitative analysis of axonal changes

Quantitative analysis of the number of axonal changes was hampered because of the variable distribution pattern of the tracer labeled fibers; thus we semi-quantitatively evaluated the degree of axonal changes using a grading system referred to a previous article with a little modification [15]. All sections from a vial containing a tracer labeled injection spot were processed to evaluate the grading of axonal changes using a motorized microscope (ECLIPSE 90i, Nikon, Japan) equipped with a Nikon cooled color CCD camera (DS-5MC-U2) and controlled by a Nikon image analysis software (NIS-Elements BR 3.1). Changes were scored separately in each brain area according to the following metric: 0 = no signs of axonal changes; 1 = sparse/slight axonal changes but less than moderate; 2 = moderate, 3–5 instances of axonal change/0.035 mm^2^ (field of view of the 40× objective lens on the microscope) only if they appeared in at least 3 different labeled fibers and were repeated in at least 3 different sections. Each instance of axonal change was scored only when swollen axons (≥1 µm in diameter) and varicosities (≥3 µm in diameter) were identified; 3 = frequent/severe, a greater than moderate amount of axonal changes. Two investigators were blind to case type during the semi-quantitative analysis.

### Western blot analysis

At 6 h, 24 h, 1 w, 2 w, 3 w or 4 w after surgery, animals were sacrificed (n = 4 per experimental condition) and then the cortex was immediately removed and stored at −86°C until use. The tissues were homogenized on ice in a Tris-Triton buffer (10 M Tris-HCl, 100 mM NaCl, 1 mM EDTA, 1% TritonX-100, 10% glycerol, 0.1% sodium dodecyl sulfate, 0.5% deoxycholate and 1 mM PMSF, pH 7.4). The total protein was isolated by centrifugation. Sample (30–50 g of protein) were electrophoresed onto a 12% SDS/polyacrylamide gel (SDS/PAGE) and transferred to nitrocellulose membranes, then blocked for 2 h at room temperature in TBS containing 0.05% Tween-20 and 5% (W/V) bovine serum albumin (BSA). Then the blot was incubated overnight at 4°C with the primary antibodies P-tau (1∶1000), Tau-5 (1∶500), AT8 (1∶2500), and β-actin (1∶500), respectively. The membranes were washed in TBS containing 0.05% Tween-20 and were incubated for 1 h at room temperature with the appropriate HRP-conjugated secondary antibody (Goat anti-rabbit IgG or Goat anti-mouse IgG, Vector, USA, 1∶8000). Blots were developed using SuperSignal West Pico Chemiluminescent substrate (Pierce, USA) in a dark chamber and were imaged by EM-CCD in a dark box. The protein bands were quantitatively analyzed using Nikon image analysis software (NIS-Elements BR 3.1). All data concerning the level of specific proteins were normalized at the level of β-actin.

### Statistical analysis

A statistical analysis of significant differences in the degree of axonal changes at the different time points between MCAO and sham group was carried out by means of Rank test. All data for mean values obtained from Western blotting were compared statistically using one-way ANOVA with the S-N-K multiple comparison method and SPSS 13.0 statistical software (SPSS Inc, Chicago, Illinois, USA). Significance level was set at P<0.05.
